# Needs Assessment for Performance Improvement of Personnel in Charge of Epidemiological Surveillance in Morocco

**DOI:** 10.1371/journal.pone.0101594

**Published:** 2014-07-07

**Authors:** Gerardo Priotto, Ahmed Rguig, Moncef Ziani, Anouk Berger, Pierre Nabeth

**Affiliations:** 1 Global Capacities, Alert & Response, World Health Organization, Lyon, France; 2 Service de Surveillance Epidémiologique, Direction d'Epidémiologie et Lutte Contre les Maladies (DELM), Rabat, Morocco; 3 Centre Hospitalier Universitaire Ibn Sina, Service de Pédiatrie, Faculté de Médecine et de Pharmacie, Université Mohammed V – Souissi, Rabat, Morocco; The Australian National University, Australia

## Abstract

**Background:**

In line with the International Health Regulations (IHR 2005), the Morocco health surveillance system has been reinforced via infrastructure strengthening and decentralization in its regions. To plan for personnel capacity reinforcement actions, a national workforce needs assessment was conducted by the National Epidemiological Surveillance Service and the World Health Organization.

**Methods:**

The assessment used an ad-hoc method comprising two stages: (1) A survey via a standardized electronic questionnaire, administered to all staff in regional and provincial surveillance teams. Data collected included demographics, basic qualification, complementary training, perceived training needs, and preferred training modalities. Individuals were asked to grade, on a nine-point scale, their perception of importance of a given list of tasks and of their capacity to perform them. The gap between perceptions was quantified and described. (2) Field visits to national, regional and provincial sites for direct observation and opinion gathering on broader issues such as motivators, barriers, and training needs from the local perspective.

**Results:**

Questionnaire respondents were 122/158 agents at 78 surveillance units countrywide. Mean age was 43.6 years and job longevity 5.7 years. Only 53% (65/122) had epidemiology training, posted in 62% (48/78) of the structures. Self-assessed capacity varied by basic qualification and by structure level (regional vs. provincial). The gap between the importance granted to a task and the perceived capacity to perform it was sizable, showing an uneven distribution across competency domains, regions, surveillance level and staff's basic qualification. From the opinions gathered, a problem of staff demotivation and high turnover emerged clearly.

**Conclusions:**

Our method was successful in revealing specific details of the training needs countrywide. A national strategy is needed to ensure rational planning of training, personnel motivation and long-term sustainability. In terms of training, an innovative program should target the specific needs per group and per region.

## Introduction

The Kingdom of Morocco is organized in regions, which are subdivided into provinces (rural) and prefectures (urban). At the regional and subregional levels, the Ministry of Health is represented by the Regional Directorate of Health and the Provincial/Prefectural Delegation of Health, respectively.

Since 1992, in order to comply with the decentralization and strengthening process launched by the Ministry of Health, the Directorate of Epidemiology and Disease Control (DELM) has established Provincial and Prefectural Epidemiology Cells (CPE), responsible for epidemiological surveillance in these administrative divisions where population ranges from 68,000 to 1,264,000 people. In 2002, Regional Observatories of Epidemiology (ORE) were established in all regions, with the objective of managing the epidemiological surveillance at regional level, taking into account regional specificities. OREs supervise the CPE's located in their respective region, and centralize the information they generate.

In 2008 the OREs became Regional Observatories of Health (ORS) and their mandate was extended to manage all of the health information for the region. At the time of this study, there were 98 structures in total (ORS plus CPE), each theoretically staffed with at least two people (a physician and a facilitator).

The decentralization was accompanied by infrastructure and equipment enhancement, telecommunications connectivity, and a series of epidemiology short training courses organized between 1996 and 2004.

All these actions aimed to strengthen the public health capacities required by the International Health Regulations (IHR), which WHO Member States have committed to implementing [1].

However, in terms of the human resources operating the system, public health officers have expressed concerns about their capacity to perform adequately and their willingness to stay for a reasonable time in the job. Indeed, it has been observed that in many resource-limited countries, decentralization has reduced the prospects for developing and maintaining skills [2].

In order to address this concern, a nation-wide assessment of epidemiology training needs of the staff in the ORS and CPE was conducted in a collaborative effort of the Ministry of Health and the World Health Organization. The objective was to inform the development of a strategy to strengthen the workforce, by (1) describing the situation of these public health officers, their perception of technical capacities and gaps, as well as their training needs; and (2) gathering opinions and suggestions from key managers and beneficiaries of the surveillance system.

## Methods

We developed an ad-hoc method inspired by methodologies previously used elsewhere [3] [4] [5] [6]. The assessment was carried out in two successive stages: (1) a standardized questionnaire administered to all staff in regional, prefectural and provincial surveillance teams, and (2) a series of interviews and field visits for direct observation and opinion gathering.

### Survey by remote questionnaire

A standardized electronic questionnaire was developed by WHO and sent by email to the staff of all ORS and CPE in the country. The distribution of questionnaires and reception of responses were centralized by the National Epidemiological Surveillance Service (SSE) within the DELM, located in the capital Rabat.

The responses were compiled in an electronic database that was subject to quality control and cleaning. Answers to open questions, such as descriptions of courses taken, were verified and standardized. Respondents were contacted individually for clarifications where necessary. The questions covered the following areas: general characteristics of personnel; training received (anytime in the past) in epidemiology, in public health, in statistics and other subjects; institutions that had provided each training; country of training; importance attached to a list of 33 tasks/activities linked to epidemiological surveillance; level of competence by task; training needs felt; and training modalities preferred.

The list of 33 tasks (Table 1) was developed on the basis of the curriculum and the evaluation results of various field epidemiology training courses in which WHO has participated. The level of competence was self-assessed by the survey participants, using the list of tasks. First, the participants were asked to rank (on a scale of 1 to 9) each task, according to its importance in the exercise of their surveillance duties. Second, the same 33 tasks were presented again and participants were asked to assess their own level of competence to perform each of them, also on a scale of 1 to 9. This scale was visually presented in three progressive stages: “weak” (values 1–3), “medium” (values 4–6), and “strong” (values 7–9).

**Table 1:** List of 33 tasks surveyed (original text in French), and their grouping in 10 domains.

Task	Domain
Calculer et interpréter les mesures de fréquence des événements de santé	Analyse descriptive
Analyser des données selon les paramètres de position et de dispersion	Analyse descriptive
Décrire les tendances des maladies selon le temps, lieu et personnes	Analyse descriptive
Déterminer des seuils épidémiques	Analyse descriptive
Etudier l'association entre une exposition et la survenue d'une maladie	Analyse recherche
Appliquer des méthodes pour contrôler les biais de sélection et d'information	Analyse recherche
Appliquer des méthodes pour contrôler les biais de confusion et les interactions	Analyse recherche
Rechercher de l'information technique et scientifique sur Internet	Analyse recherche
Présenter des données avec des tableaux et graphiques	Comm scientifique
Préparer des rapports de surveillance épidémiologique et de retro-information	Comm scientifique
Communiquer oralement à l'aide de supports visuels	Comm scientifique
Rédiger un article scientifique	Comm scientifique
Décrire les nouveautés introduites par le RSI 2005	Connaissance RSI
Respecter l'éthique et confidentialité dans les activités de santé publique	Ethique
Utiliser des outils informatiques de collecte et d'analyse de données	Informatique
Construire une définition de cas	Investig. Epidémie
Calculer et interpréter la sensibilité et spécificité d'une définition de cas ou d'un test	Investig. Epidémie
Investiguer une épidémie de façon méthodique	Investig. Epidémie
Rédiger un rapport d'investigation d'épidémie	Investig. Epidémie
Appliquer les procédures correctes de collecte et de transport de prélèvements biologiques	Investig. Epidémie
Réaliser des enquêtes de couverture vaccinale	Investig. Epidémie
Déterminer l'efficacité vaccinale	Investig. Epidémie
Contribuer à la préparation aux épidémies	Riposte
Recommander des mesures de contrôle et prévention des épidémies	Riposte
Contribuer à la riposte en tant que spécialiste en surveillance	Riposte
Contribuer à l'élaboration des plans de communication en situation d'urgence	Riposte
Formuler des lignes directrices pour l'information au public en cas de risque réel ou potentiel de maladie infectieuse	Riposte
Préparer un plan de supervision d'un système de surveillance	Supervision/évaluation
Réaliser une supervision des acteurs de la surveillance	Supervision/évaluation
Evaluer un système de surveillance	Supervision/évaluation
Mettre en place un système de surveillance épidémiologique en suivant les étapes adéquates	Syst. de Surveillance
Faire des choix rationnels entre surveillance exhaustive et sentinelle, et/ou entre surveillance passive et active	Syst. de Surveillance
Etablir les mécanismes de collecte de données	Syst. de Surveillance

Because both parameters were measured with the same scale and by the same person, the calculation of the difference between the two figures provided a direct measurement of the gap between the importance attached to a given function and the perceived level of competence for that same function. The magnitude of this gap helped identify where a lack of competence was felt as a priority.

The analysis consisted of quantitative description of data, stratifying by area of expertise, basic qualification (degree or diploma giving a professional status pertinent to the job), type of structure (ORS or CPE), and by administrative divisions (region and province/prefecture). Mapping (not shown in this manuscript) was used to present the geographical distribution of certain characteristics.

**Figure 1:**
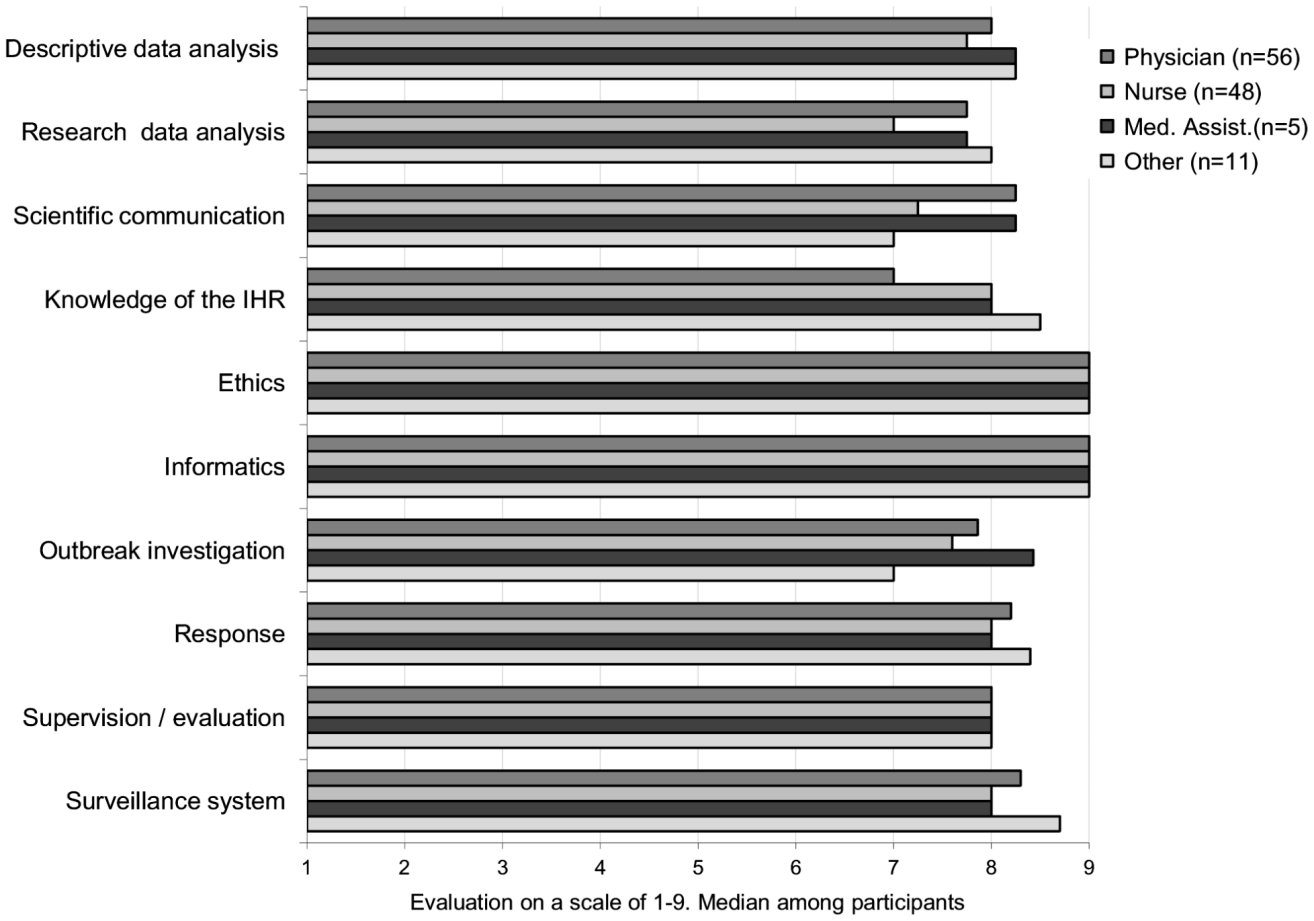
Importance attributed to surveillance tasks (grouped in 10 domains), by basic qualification, Morocco 2011.

**Figure 2:**
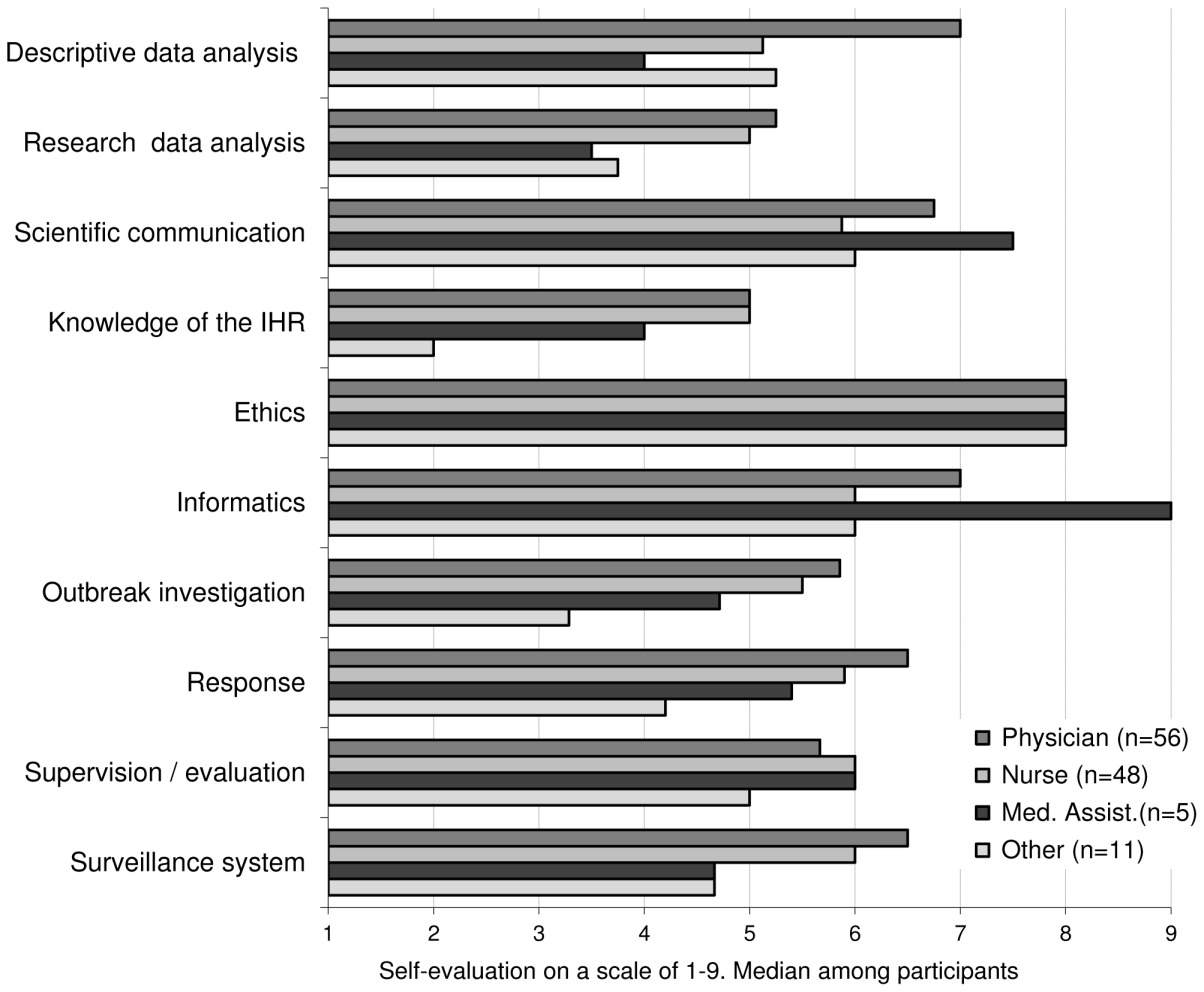
Level of self-estimated competence in each area, by basic qualification, Morocco 2011.

**Figure 3:**
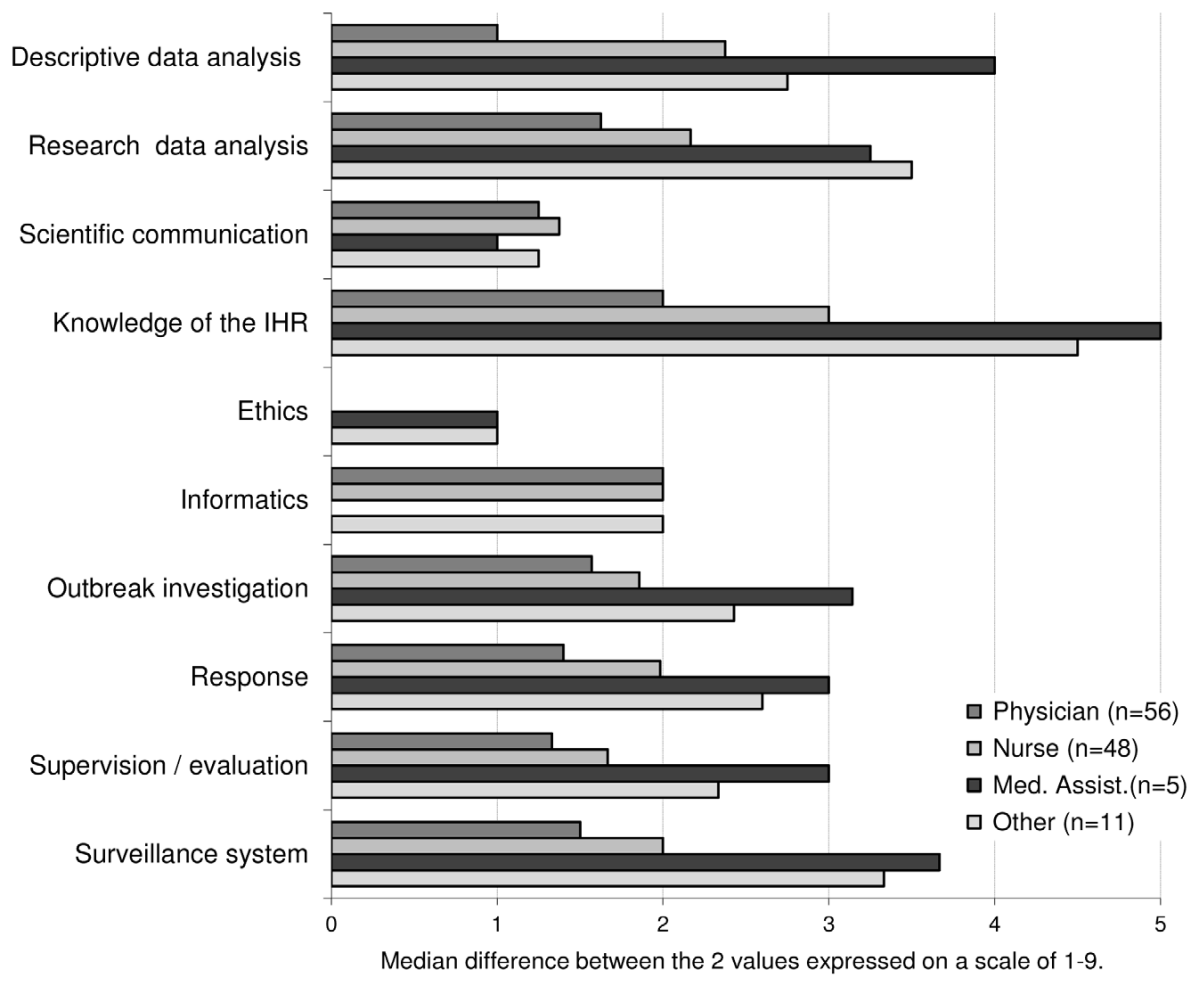
Competence gap (self-rated), by basic qualification. Gap between the importance given to surveillance tasks (grouped in 10 broad areas) and the self- estimated competence (rated by each participant using a scale of 1 to 9), by basic qualification categories.

Responses to the 33 tasks related to surveillance were synthesized for analysis by grouping them into 10 domains, as shown in Table 1 and Figures 1, 2 and 3.

Data analysis was performed with EpiInfo 3.5.4 and Stata 10 software.

### Interviews and field visits

Interviews and field visits were organized with the objectives of (1) gathering information and opinions of key players on broader issues such as motivators and barriers, and on training needs from the local perspective; and (2) directly observing and discussing the methods and tools used for the surveillance activities.

At central level, the interviewees included relevant officials of the Ministry of Health's directorates which are involved in public health surveillance and the health information system, as well as epidemiology training providers. In each region visited we interviewed the Regional Health Director as well as the technical personnel conducting surveillance tasks at the ORS and at least at one CPE. For the field visits we selected five regions with different performance levels (appraised by the DELM on the basis of recent experience), in order to obtain representative results, especially regarding the inter-region variability. The visits and interviews were prepared on the basis of the results of the questionnaire, the review of documents and tools used for public health surveillance in Morocco, and the results of other consultations carried out in the past 10 years on related issues. Interviews were conducted in person and documented verbatim. A semi-structured interview guide included questions pertaining to interviewees' expectations regarding the output of the surveillance system, their working experience with the surveillance units that had revealed capacity gaps, their beliefs about the nature of existing barriers, and their beliefs about training modalities that would be most effective. Probing questions were used where necessary to seek comprehensive data.

Qualitative data from the interviews and field observations were analyzed manually with a qualitative thematic approach, without coding or quantitative transformation, looking for deeper meaning in the individual expressions and observations. Salient ideas stemming from different sources were triangulated to identify areas of convergence. The convergent ideas were initially documented by type of source in the study report. In this manuscript they are further consolidated, and presented in 3 distinct theme categories (Training Needs, Training Modalities, and Other Issues Concerning Human Resources).

## Results

### Results of the survey by remote questionnaire

In November 2010 the electronic questionnaire was sent by e-mail to all 158 staff located at functioning health surveillance structures. At the time there were 8 CPE without staff. The transmission of the questionnaires and the responses was confirmed as successful, rapid, and cost-free. Between that date and January 2011, 122 responses to the questionnaire were obtained from 78 structures. All these questionnaires were answered completely: no questions were left unanswered. The distribution of respondents by region was uneven, with 51% concentrated in five regions (Rabat-Salé-Zemmour-Zaër, Oriental, Souss-Massa-Draa, Tanger-Tétouan and Fez-Boulemane). The mean age was 43.6 years and the mean seniority in the current position was 5.7 years (Table 2). Overall, the ORS staff had less seniority in their job than the CPE staff (mean 4.2 vs. 6.3 years), although their age was not different. Basic qualifications within the ORS teams were more diverse, sometimes composing multidisciplinary teams with rich skill mixes.

**Table 2:** General characteristics of survey participants, Morocco 2011.

		ORS^a^ (N = 35)			CPE^b^ (N = 87)			Total (N = 122)		
		n	value	min-max	n	value	min-max	n	value	min-max
Demographics	Age in years, mean	34	43.7	[26–56]	87	43.5	[24–57]	121	43.6	[24–57]
	Sex ratio M:F	35	1.3		87	4.8		122	3.1	
	Seniority in years, mean	34	4.2	[1–12]	85	6.3	[0–33]	119	5.7	[0–33]
Basic qualification	Physician	18	51.4%		39	44.8%		57	46.7%	
	Medical Assistant	3	8.6%		2	2.3%		5	4.1%	
	Registered Nurse	7	20.0%		42	48.2%		49	40.2%	
	Other^c^	7	20.0%		4	4.6%		11	9.0%	

a ORS: Observatoire Régional de Santé;

b CPE: Cellule Provinciale ou Préfectorale d'Epidémiologie;

c Other basic qualifications (in decreasing frequency): engineer, hygiene technician, administrator, biologist.

Only 53% (65/122) of the staff had epidemiology training and they were posted in 62% (48/78) of the surveillance structures. The epidemiology training received by staff was unevenly distributed across the regions: of a total of 92 courses in which the respondents had participated, most of the participation was concentrated in the regions of Fez-Boulemane, Souss-Massa-Draa and Tanger-Tétouan (median of 12 epidemiology courses followed by the team, range 11 to 14), while on the other extreme, in 5 other regions a median of 1 course had been followed by the whole team (range 0 to 2).

Of the surveillance tasks presented in the questionnaire, all 33 tasks were perceived as very important by all staff, both at the ORS and the CPE teams (the median score ranged from 7 to 9). The most frequently highlighted were the tasks in the areas of ethics and computing (general median score 9 for both). Outbreak investigation was seen as relatively less important (general median 7.7). Knowledge of the International Health Regulations (IHR) was seen as less important for staff at the CPE's (CPE median 7).

The majority of staff considered they did not have an adequate level of competence in most areas. The areas of IHR, outbreak investigation and analytical methods applied to research, stood out as particularly weak.

The comparison across basic qualification groups (Figure 2) revealed substantial differences by area of expertise and by basic qualification, which exposed key details of the training needs. For example, physicians graded themselves higher than others in most areas, except in the supervision and evaluation areas. The competence levels of medical assistants were often discordant with that of doctors and nurses: higher in scientific communication and computing, while lower in data analysis, investigation and surveillance system operation.

The self-assessed level of competence also showed disparities across regions (data not shown).

The gap between the importance granted to a task and the individual capacity to perform it was of considerable magnitude for the majority of respondents. The overall difference between the two parameters, which were measured on a 9-points scale, showed a median of 1.7 points. A marked disparity was seen between the basic qualification groups: physicians exhibited smaller gaps (magnitude of 0–2 points) than the group of medical assistants and “others” (1–5 points), while the group of nurses was in an intermediate situation (1–3 points). The magnitude of the gap varied per type of task as well (Figure 3).

When participants were asked (in an open-ended question) to name areas of knowledge where they felt the principal need for training, a majority (82.8%) pointed to Epidemiology (where the most cited spontaneous categorizations were “field”, “general” and “surveillance” epidemiology), followed by Biostatistics (58.2%) and Informatics (49.2%).

Regarding the preferred learning modalities, of the following four choices presented, the large majority (91.4%) opted for the face-to-face courses, followed by self-learning compact disks (59.4%), online courses with remote tutoring (52.9%) and self-learning online courses (51.6%).

We explored the possible associations between the capacity gap and the distance of a duty station from the capital, Rabat, but the distance factor alone did not show a clear trend.

### Results of the interviews and field visits

These activities were conducted in January 2011. The interviews carried out with high-level officers and team members included the following governmental institutions: Directorate of Epidemiology and Disease Control (DELM), Directorate of Hospitals and Outpatient Care (DHSA), National Institute of Hygiene (INH), National Institute of Health Administration (INAS), Division of Computer Science and Methods (DIM), Directorate of Planning and Financial Resources (DPRF), Communication Division (DIV.COM), and Poison and Pharmacovigilance Center (CAPM).

We carried out field visits in the following five regions: Gharb-Chrarda-Beni-Hassen, Grand Casablanca, Chaouia-Ouardigha, Tanger-Tétouan, and Rabat-Salé-Zemmour-Zaër. In total, 5 regional directors (or acting directors) were interviewed, 5 ORS and 7 CPE were visited. Interviewees from different institutions and at different levels provided their own perspective stemming from their particular position and experience. We found no contradictory views. Rather complementary perceptions with a high degree of convergence were established.

The most common observations are reported hereafter, grouped in three thematic categories.

#### Training Needs

The skills level is very uneven among staff. Due to the high turnover, many of those who are currently in office have not received training specific to their duties. Training programs attended by the ORS and CPE staff are often inadequate for their actual functions. Training courses taken abroad are generally based on contexts, examples and needs different from those of Morocco.

There is need for training that is better adjusted to the type of surveillance structure (ORS or CPE), as responsibilities are different (e.g. data management at CPE's is limited to epidemiological data while the ORS's deal with all health information). ORS teams need specific training on certain national health information tools, such as the National Health Information System (SNIS) and the Health Care Facilities Databank (BOSS). Training would have greater impact if it was more focused on specific regional health issues.

Training supply has not been constant over time, and current training programs are insufficient, in terms of output and specificity, to address the needs of the surveillance workforce at all levels.

The areas of knowledge prioritized by interviewees included: informatics, data management and analysis (in particular for surveillance functions), basic epidemiology and surveillance methods, early warning systems, scientific communication, risk communication, feedback communication, supervision methods and remediation of completeness and timeliness problems. The ORS teams put forward some additional priority needs, pertinent to their responsibilities: planning and management, monitoring and evaluation of surveillance systems, data analysis for non-communicable diseases, research methods, and geographic information systems.

#### Training Modalities

Interviewees offered a number of suggestions regarding the design of training programs: to avoid grouping together individuals with different qualification profiles (physicians, medical assistants, nurses, etc); to plan the immediate application of the skills acquired in the trainee's job, under tutoring; to adapt training contents to the specific needs; to avoid dense theory and exercises without practical application; to decentralize training in regions; to link training to the organization/participation in scientific conferences; to streamline the organization of training in a way that minimizes the time taken on the staff's activities.

#### Other issues concerning human resources

Surveillance staff lack motivation, which leads to high turnover and understaffing. Several factors were mentioned: their routine is mostly limited to data collection without analysis and publication, a poor career progression, poor visibility of their role and the fruits of their work, and professional isolation.

Staff tend to stay a short time in office, and many that depart (mostly via transfers to positions in different health sectors) are not replaced because potential candidates do not feel attracted to these jobs.

Most of the training courses completed by staff were not officially recognized and did not have an impact on career progression.

Staff feel disconnected from peers carrying out similar work nationally and internationally. They lack incentives and opportunities to present their work and to learn from others.

## Discussion

The methods used in this assessment allowed for the collection of quantitative and qualitative data that, combined, provided a fairly comprehensive insight of the training needs and other important elements that impact the strength of the workforce. The use of an electronic questionnaire that could be successfully transmitted to all staff turned out an effective method to obtain country-wide standardized information in a very short time and at little cost. Having analyzed these data prior to the field visits was useful in selecting the regions to visit and in structuring the interviews for an optimal exploration of key issues.

Our findings confirmed the concerns of national public health officers: that an important weak point in the Moroccan surveillance system is the lack of appropriate human resources, both in number and in skills. Throughout the national network, many surveillance facilities are understaffed, and some are even totally vacant. There appears to be a need for redistribution over the territory, although this point requires further analysis by the health authorities. The distribution of staff should be guided by the interplay of several factors, including the relative workload, the local constraints, the mapping of public health risks, the strategic priorities and the administrative and socio-political constraints.

The design of this study did not include the search for factors influencing the capacity gap in a given health surveillance structure, and our exploratory analysis of the distance factor did not reveal this variable as a stand-alone proxy. A wide-ranging, multivariate analysis would be needed to unveil the contributing factors in the context of Morocco. This type of analysis would be best done via a quantitative study with planned endpoints and outcome measures, using a representative sample with a size sufficient for multivariable methods. Such study can be informative for fine-tuning the training curricula and the human resources policy, but should not stand in the way of immediate action, as at this stage many key issues have been identified with a large consensus, providing leads on which to work.

We recommend the formulation of a national strategy to ensure the rational planning of training, the high motivation of personnel, and the long-term sustainability of performance. Because the issue of the health workforce is complex, comprising many actors and stakeholders [7], one of the conditions for success of such a strategy is the involvement of high-profile decision-makers, not only of the health sector, but of all other sectors that play a role, and that would eventually be required to contribute. The application of this strategy is to be followed and adjusted over time by the same actors that conceived its design.

The skill level of surveillance staff throughout the national network is highly variable. Launching a new training program appears necessary, which should have a larger coverage than the current programs, and should target the specific needs per professional group and per type of surveillance facility. In coherence with the current national policy for public health, training should be decentralized in order to better tailor the methods and contents to the needs in each region. With this approach, an additional advantage expected is the minimization of the time for which the trainees would be absent from their duties. On the other hand, expected disadvantages may include overall time to achieve and/or financial outlay. As suggested by many of the interviewees in this study, the contents and modalities of training activities would be better adapted to the needs if they were developed with the participation of representatives from the target audience.

The observed continual loss of human resources in the sector appears to be associated with low motivation to stay in the job and difficulty filling vacancies that are not attractive to the appropriate candidates. It is clear, therefore, that another type of action, beyond training, is needed to strengthen the workforce in a sustainable way. This action, as found in this assessment, could involve measures to improve the formal recognition of training accomplished by staff, the clarity of career paths, the visibility and valorization of staffs' contribution to public health, and the participation in professional peer networks and activities.

Performance does not depend solely on the individual workers' knowledge and competencies, but also on the work environment, which may pose, in reality, the greatest barriers. It is important that a performance improvement strategy takes into account a comprehensive model encompassing both the individual elements (knowledge/skills, capacity, motives) and the environmental elements (information, resources, incentives) [8]. Such a systematic approach is key to determining the underlying causes of the performance gaps, and for targeting specific improvements with a high impact.
